# First Live-Experience Session with PET/CT Specimen Imager: A Pilot Analysis in Prostate Cancer and Neuroendocrine Tumor

**DOI:** 10.3390/biomedicines11020645

**Published:** 2023-02-20

**Authors:** Lorenzo Muraglia, Francesco Mattana, Laura Lavinia Travaini, Gennaro Musi, Emilio Bertani, Giuseppe Renne, Eleonora Pisa, Mahila Esmeralda Ferrari, Uberto Fumagalli Romario, Ottavio De Cobelli, Nicola Fusco, Francesco Ceci

**Affiliations:** 1 Division of Nuclear Medicine, IEO European Institute of Oncology IRCCS, 20141 Milan, Italy; 2 Division of Urology, IEO European Institute of Oncology IRCCS, 20141 Milan, Italy; 3 Department of Oncology and Hemato-Oncology, University of Milan, 20141 Milan, Italy; 4 Division of Digestive Surgery, IEO European Institute of Oncology IRCCS, 20141 Milan, Italy; 5 Division of Pathology, IEO European Institute of Oncology IRCCS, 20141 Milan, Italy; 6 Unit of Medical Physics, IEO European Institute of Oncology IRCCS, 20141 Milan, Italy

**Keywords:** PET/CT, PET, PET/CT specimen imager, radio-guided surgery

## Abstract

Objective: to evaluate the feasibility of the intra-operative application of a specimen PET/CT imager in a clinical setting. Materials and methods: this is a pilot analysis performed in three patients who received an intra-operative administration of ^68^Ga-PSMA-11 (n = 2) and ^68^Ga-DOTA-TOC (n = 1), respectively. Patients were administrated with PET radiopharmaceuticals to perform radio-guided surgery with a beta-probe detector during radical prostatectomy for prostate cancer (PCa) and salvage lymphadenectomy for recurrent neuroendocrine tumor (NET) of the ileum, respectively. All procedures have been performed within two ongoing clinical trials in our Institute (NCT05596851 and NCT05448157). Pathologic assessment with immunohistochemistry (PSMA-staining and SSA immunoreactivity) was considered as standard of truth. Specimen images were compared with baseline PET/CT images and histopathological analysis. Results: Patients received 1 MBq/Kg of ^68^Ga-PSMA-11 (PCa) or 1.2 MBq/Kg of ^68^Ga-DOTA-TOC (NET) prior to surgery. Specimens were collected, positioned in the dedicated specimen container, and scanned to obtain high-resolution PET/CT images. In all cases, a perfect match was observed between the findings detected by the specimen imager and histopathology. Overall, the PET spatial resolution was sensibly higher for the specimen images compared to the baseline whole-body PET/CT images. Furthermore, the use of the PET/CT specimen imager did not significantly interfere with any procedures, and the overall length of the surgery was not affected using the PET/CT specimen imager. Finally, the radiation exposure of the operating theater staff was lower than 40 µSv per procedure (range 26–40 μSv). Conclusions: the image acquisition of specimens obtained by patients who received intra-surgery injections of ^68^Ga-PSMA-11 and ^68^Ga-DOTA-TOC was feasible and reliable also in a live-experience session and has been easily adapted to surgery daily practice. The high sensitivity, together with the evaluation of intra-lesion tumor heterogeneity, were the most relevant results since the data derived from specimen PET/CT imaging matched perfectly with the histopathological analysis.

## 1. Introduction

Positron emission tomography (PET) imaging is considered one of the leading imaging techniques to investigate oncological patients and it is considered the standard of care in many solid tumors and hematological malignancies [1]. Recently, several technological improvements appeared in the field of PET imaging, ranging from digital detectors (e.g., silicon photomultipliers) to long axial field-of-view scanners [2]. However, the attention moved also to smaller PET/computed tomography (CT) tomographs that might be implemented in the daily clinical practice of surgical oncology. Recently, a compact and mobile PET/CT specimen imager was developed to obtain full 3D high-resolution images, assisting surgeons and pathologists during the surgical procedure directly in the operating theater. This specimen imager combines the picomolar sensitivity of PET (at sub-millimeter resolution) with anatomical information obtained from a high-resolution micro-CT. The first research application was in breast cancer using ^18^F-FDG as a PET radiopharmaceutical, where a multicenter clinical trial is currently ongoing (NCT04999917). Intra-operative high-resolution PET/CT imaging has shown promising results for margin assessment in breast malignancies, where a respective sensitivity and specificity of 84% and 96% was reached for the identification of positive margins of the invasive component of invasive ductal carcinoma [3]. Explorative studies in pancreatic adenocarcinoma and in head and neck cancers have also demonstrated the feasibility of intra-operatively imaging radiotracer uptake in high-resolution specimen PET/CT images [4,5].

Recently, novel radiopharmaceuticals emerged as game changers in prostate cancer (PCa) with prostate-specific membrane antigen (PSMA) PET and in neuroendocrine tumor (NET) with somatostatin receptor (SSR) based PET. PSMA-PET showed its superiority compared to conventional imaging or other PET tracers in randomized clinical trials [6,7] or registry trials [8,9,10] to stage Pca or in case of disease recurrence, while SSR-agonist PET, is currently the standard of care to investigate gastro-entero-pancreatic NET (GEP-NET), especially in case of low-grade disease (G1-G2) [11]. Furthermore, both PSMA inhibitors and SSR agonists are theranostic agents and can be labeled with isotopes for PET imaging (e.g., Gallium-68) or high-energy beta or alpha emitters (e.g., Lutetium-177 or Actinium-225) for radio-ligand therapy. Therefore, there is rising and increasing interest to explore the potential of PET/CT specimen imagers beyond ^18^F-FDG, and applying this high-resolution imaging in patients undergoing surgery for Pca or NET.

The aim of this pilot analysis was to assess the feasibility of a PET/CT specimen imager in a live-experience session in patients who received an intraoperative injection of ^68^Ga-PSMA-11 and ^68^Ga-DOTA-TOC.

## 2. Materials and Methods

### 2.1. Patient Population

This pilot analysis was performed as a secondary objective of two trials currently ongoing in our Institute in Pca (NCT05596851) and NET (NCT05448157) designed to evaluate the safety and the efficacy of a positron probe detector (DROP-IN β-probe) for live intra-operative radio-guided surgery. Three patients enrolled in these trials have been considered eligible for this supplemental analysis, two affected by Pca and one affected by NET of the ileum. In all cases, patients received a baseline whole-body PET/CT to assess the burden of the disease (^68^Ga-PSMA-11 PET/CT and ^68^Ga-DOTA-TOC PET/CT, respectively). The scans were performed in the Division of Nuclear Medicine of our Institute (GE Discovery MIDR) according to international procedural guidelines, 4–6 weeks prior to surgery. All patients signed informed consent form prior to enrollment and studies have been approved by local ethical committee (Comitato Etico degli IRCCS – Istituto Europeo di Oncologia e Centro Cardiologico Monzino) and scientific review board. The study has been approved by local ethical committee and scientific review board (Trial Identifier ID Code IEO-1703; NCT05596851; submitted 24 October 2022. Trial Identifier ID Code IEO-1740; NCT05448157; submitted 1 July 2022).

### 2.2. Specimen PET/CT Imager

**Figure 1:**
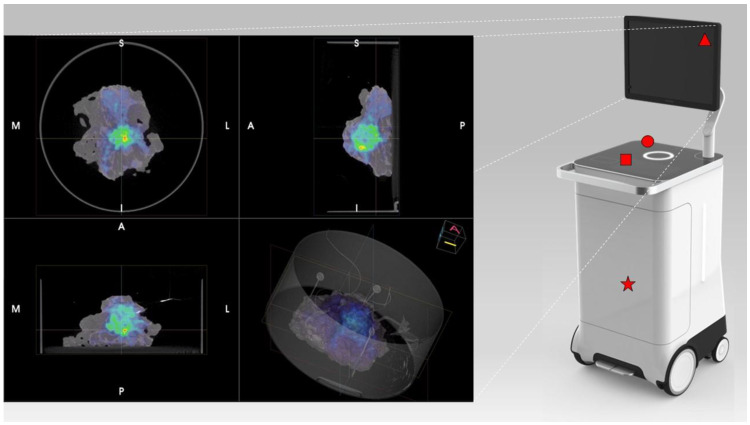
Overview of the PET/CT specimen imager. On the right is the device with its main constitutes: the medical display (red triangle), the specimen box container (red circle), the viewer interface (red square), and the hardware that allows a two-step acquisition (CT and PET—red star). The viewer of the PET/CT specimen imager shows the images in three different planes (coronal, sagittal, and transversal) as shown in the left figure. The user can scroll through each of the planes through a dedicated viewer interface (red square). As such the viewer does provide a 3D image of the specimen. Additionally, a 3D render is present, which can be rotated by the user.

PET/CT images were obtained using the AURA10^®^ Specimen PET/CT imager (Xeos Medical NV, Ghent, Belgium). Subsequently, optical, micro-CT and micro-PET images were acquired. Specimens were scanned in a dedicated specimen container (10 cm diameter and 4 cm height) (Figure 1). CT images were reconstructed using the Image Space Reconstruction Algorithm at 100 µm voxel size. PET images were reconstructed using 20 iterations of the ordered subset expectation maximization algorithm at 400 µm voxel size. The total duration between specimen insertion and visualization of the images on the device was 10 min.

### 2.3. Description of the Surgery Procedure

This analysis was a secondary objective of two ongoing phase II trials at our institution (NCT05596851; NCT05448157), involving the intra-operative administration of ^68^Ga-PSMA-11 and 68Ga-DOTATOC for radio-guided surgery with a dedicated beta-probe. Therefore, the activity used is defined by the protocol of the trials. In this pilot study, we demonstrated that high-quality images can be made with the specimen PET/CT imager, using a low activity of ^68^Ga-PSMA-11 and ^68^Ga-DOTATOC. The PCa patients received 1 MBq/kg of ^68^Ga-PSMA-11 and the NET patient received 1.2 MBq/kg of ^68^Ga-DOTA-TOC. PET radiopharmaceutical administration was performed directly in the surgery theater at the beginning of the procedure. For Pca patients a robotic-assisted laparoscopic approach was adopted. Extended pelvic lymph node dissection (ePLND) was performed according to a standard template, including internal/external iliac nodes, obturator nodes, and common iliac ones. Radical prostatectomy (RP) was performed after ePLND, according to the study protocol (NCT05596851). For the ileum GEP-NET patient, open surgery was chosen as surgical approach, according to study protocol (NCT05448157).

In all cases, surgical specimens were collected in the operating theater and placed in dedicated containers by the pathologist, who oriented the specimens in the same position adopted for histopathological analysis. Specimen containers were placed in the PET/CT specimen imager and one scan was performed for each specimen. PET/CT acquisitions were performed 2.5 to 4.5 h post-injection. Such delayed acquisition times can be made due to the high detection sensitivity of the imager. Of some specimens, several acquisitions were performed, to explore the potential of administering less activity. High-resolution fused 3D images were obtained and reviewed in a multidisciplinary setting involving nuclear medicine physicians, surgeons, and pathologists. Pathologic assessment with immunohistochemistry (PSMA-staining and SSA immunoreactivity) was considered as standard of truth. Specimen images were subsequently compared with baseline PET/CT images and histopathological analysis. 

Baseline whole-body PET/CT images were performed according to EANM guidelines after intravenous injection of 2 MBq/kg of ^68^Ga-PSMA11 and 2,5 MBq/kg of ^68^Ga-DOTATOC. Baseline scans were performed within six weeks before the day of the surgery (patient 1: 4 weeks; patient 2; 6 weeks; patient 3: 4 weeks).

## 3. Results

**Figure 2:**
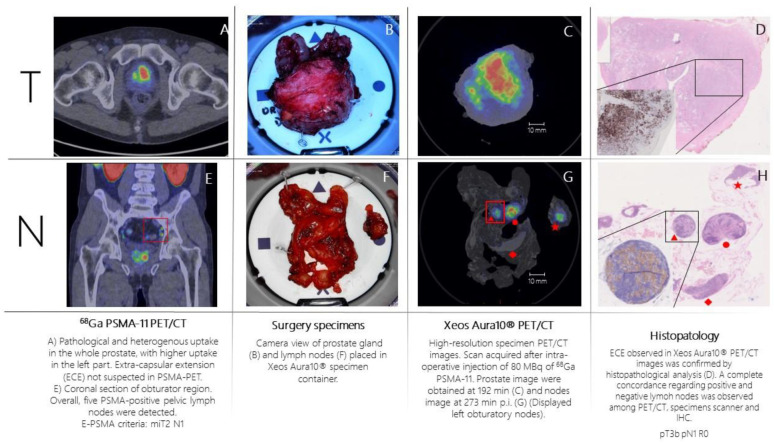
Patient 1 (prostate cancer): ^68^Ga-PSMA-11 whole-body PET/CT image showed pathological PSMA uptake (high expression, E-PSMA Visual Score = 3) in the prostate (**A**) and in five pelvic lymph nodes (**E**). This figure also represented surgery specimen’s macro pictures (**B**,**F**), high-resolution specimen PET/CT images (**C**,**G**) and histopathological analysis in the specimens where immunohistochemical analysis (PSMA staining) was performed (see correspondence between red shapes in subfigures (**G**,**H**)). Red shapes explanation same as in Figure 1.

Patient n.1 was referred to PSMA-PET (Figure 2A,E) for high-risk Pca (Gleason Score = 4 + 4; biopsy, 6/6 positive cores bilateral, initial PSA = 10 ng/mL). PSMA-PET showed pathological and heterogeneous uptake diffusely comprising the left part of the prostate, without suspicion of extra-capsular extension. Additionally, five PSMA-positive pelvic lymph nodes were detected. According to E-PSMA reporting criteria [12] the patient was considered miT2N1M0. On the day of surgery, 80 MBq of ^68^Ga-PSMA-11 was administered intravenously. The prostate gland and pelvic lymph nodes were resected, and all specimens were scanned in the PET/CT specimen imager. Scans were performed sequentially, once the specimens were positioned in the container and properly oriented by the pathologist, at 192 min (prostate) and 273 min (lymph nodes) post-injection. High-resolution PET/CT images (Figure 2C,G) showed higher spatial resolution compared to conventional PET images. This indicates a potential for intraoperative margin assessment, complementary to pathological analysis. The left basal margin’s involvement was strongly suspected in the prostate gland image (Figure 2C,D). All resected lymph nodes were scanned. Histopathology analysis was performed and compared with the specimen PET/CT images (Figure 2D,H). For the prostate gland, the areas of PSMA-uptake in the micro-PET image corresponded to prostate cancer lesions on histopathological analysis. Extra-prostatic extension, as suspected from the micro-PET images, was also confirmed. Lymph nodes that showed PSMA uptake on micro-PET were confirmed to contain metastatic tumor cells on histopathological analysis. All positive lymph nodes detected in preoperative whole-body PET/CT were resected and imaged.

**Figure 3:**
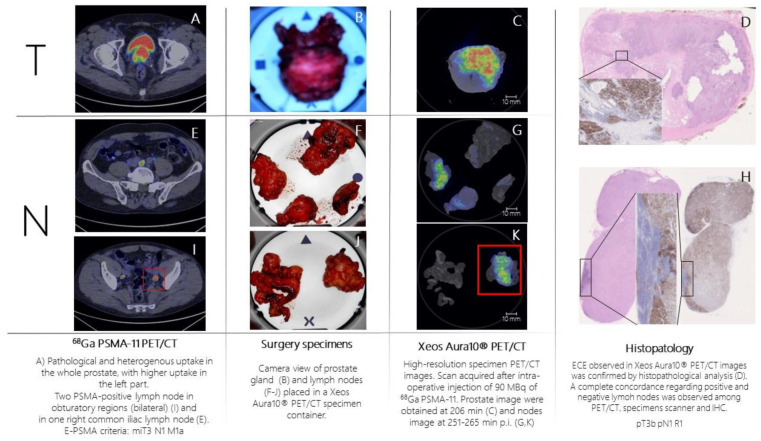
Patient 2 (prostate cancer): ^68^Ga-PSMA-11 whole-body PET/CT images showed pathological PSMA uptake (high expression, E-PSMA Visual Score = 3) in the prostate (**A**) and in three pelvic lymph nodes (**E**,**I**). This figure also represented surgery specimen’s macro pictures (**B**,**F**,**J**), high-resolution specimen PET/CT images (**C**,**G**,**K**) and histopathological analysis in the specimens where immunohistochemical analysis (PSMA staining) was performed (**D**,**H**).

Patient n.2 was referred to PSMA-PET (Figure 3A,E,I) for high-risk Pca (Gleason Score = 4 + 5; fusion biopsy, 21/21 positive cores bilateral, initial PSA = 8 ng/mL). PSMA-PET showed pathological and heterogeneous uptake in the whole prostate, with suspicion of extra-capsular extension, two PSMA-positive pelvic lymph nodes, and one PSMA-positive extra-pelvic lymph node. According to E-PSMA reporting criteria [12] the patient was considered miT3N1M1a. On the day of surgery, 90 MBq of ^68^Ga-PSMA-11 was administered intravenously. The prostate gland and lymph nodes were resected (Figure 2B,F,J) and all specimens were scanned in the PET/CT specimen imager. Scans were performed sequentially, once the specimen was positioned in the container and properly oriented by the pathologist, at 206 min (prostate) and 265 min (lymph nodes) post-injection. Images (Figure 3C,G,K) showed a high spatial resolution, and margin involvement was strongly suspected (Figure 3C,D), in accordance with baseline PSMA-PET. PET/CT specimen images offered a high-resolution view of the distribution of PSMA inside the prostate lesion, highlighting the high heterogeneity of PSMA expression in the prostate. As in the first case, areas of PSMA-uptake in the micro-PET image corresponded to prostate cancer lesions on histopathological analysis, and a positive margin—as suspected from the microPET-CT images—was confirmed. Lymph nodes that showed PSMA uptake on micro-PET were confirmed to contain metastatic tumor cells on histopathological analysis.

**Figure 4:**
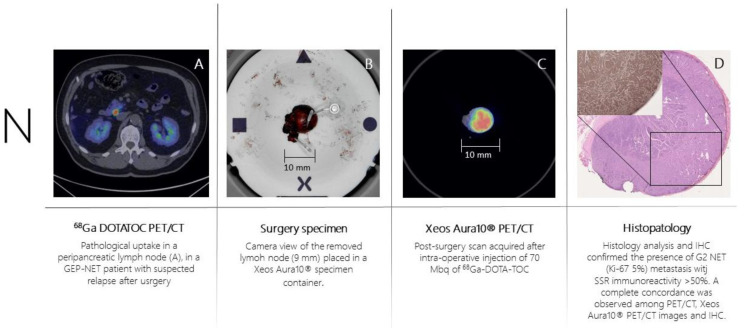
Patient 3 (GEP-NET): ^68^Ga-DOTA-TOC whole-body PET/CT images showed pathological DOTA-TOC uptake in one single peripancreatic lymph node (**A**,**B**). High-resolution specimen PET/CT images confirmed the positivity (**C**) and the presence of metastasis from GEP-NET was confirmed by histopathological analysis of the specimen (**D**).

Patient n.3 underwent 68Ga-DOTA-TOC-PET (Figure 4A) for recurrent ileum GEP-NET (G2; Ki-67 = 5%). The PET scan showed the presence of a peri-pancreatic lymph node suspected of disease location. On the day of surgery, 70 MBq of ^68^Ga-DOTA-TOC was administered intravenously. The PET-positive lymph node was resected and placed in the PET/CT specimen container. The specimen was scanned in the PET/CT specimen imager (Figure 4B,C) at 150 min post-injection properly showing a high-resolution view of homogeneous ^68^Ga-DOTA-TOC biodistribution within the lymph node. The specimen was analyzed for histopathology and immune histochemistry assessment with SSR immune reactivity (Figure 4D). A specipin was positioned in a region with high radiotracer uptake in the specimen as seen on the high-resolution PET/CT images. The positioning of this pin guided the pathologist to slice the specimen in the region of interest. This allowed an accurate comparison between the ^68^Ga-DOTA-TOC uptake and the histopathological SSR immune reactivity. All three methodologies (baseline PET/CT, specimen PET/CT, and histopathology) were fully concordant, and the lymph node was considered a true positive finding.

Table 1 and Table 2 summarize all semi-quantitative data about radiopharmaceuticals uptake, administered activity, and acquisition time both in whole-body PET/CT and high-resolution PET/CT specimen imaging.

Finally, in all procedures, the radiation exposure of the operating theater staff located near the surgical table was measured using electronic personal dosimeters (Thermo Scientific™ EPD Mk2™) and the absorbed dose was below 40 μSv (range 26–40 μSv) for each procedure.

## 4. Discussion

In this pilot analysis, first, we assessed the feasibility of integrating this new-generation imaging approach in a live surgery session. The collection and the scan of the specimens did not significantly interfere with any procedure, and the overall length of the surgery was not affected using the PET/CT specimen imager. Second, the radiation exposure generally measured for radio-guided surgery using gamma emitting isotopes (e.g., ^99m^Tc or ^18^F) is low and this technique is extensively used in clinical practice. With ^99m^Tc radiation doses per procedure to the surgeon’s chest are comprised between 0.2–0.8 µSv per MBq of activity present at the time of surgery, while for ^18^F radiation exposures to the surgeon’s chest is comprised between 10 to 217 µSv for procedures. The radiation exposure for the operating theater staff measured in our pilot analysis was low as well (less than 40 μSv; range 26–40 μSv), confirming the feasibility of injecting positron emitting isotopes (e.g., ^68^Ga or 18F) directly in the surgery theater. Finally, the specimen PET images were reliable, and in full concordance with the information derived by the histopathological analysis. Moreover, they had a higher spatial resolution compared to conventional PET images. The extraction of semiquantitative parameters (e.g., SUVmax or metabolic tumor volume) was also feasible.

At present, the implementation in clinical practice of this device has not been broadly explored. However, the development of PET/CT specimen imagers has a promising perspective and embraces the integration of new-generation imaging with the most updated surgery techniques. This new approach might improve surgeons’ confidence in ensuring the complete removal of the primary tumor. Moreover, the precise correspondence of receptor-target radiopharmaceutical uptake and immuno-histochemistry staining confirmed the precision of this imaging procedure to evaluate intra-lesion heterogeneity. Thus, the high spatial resolution of the PET specimen imager offers a deeper understanding of the bio-distribution pattern of the radiotracers inside cancer lesions. In this scenario, the use of a specimen imager might be an adjunct tool for the pathologist to better optimize resources and for a “live” assessment of specific tumor biomarkers directly during surgery.

Another potential advantage might be related to the high negative predictive value (NPV) of this imaging procedure. In patients 1 and 2, histopathology confirmed the lymph node showing high PSMA expression as true positive findings. However, PSMA-negative nodes were also included in the specimen container. The histopathological analysis confirmed the lack of malignant cells in these lymph nodes, thus resulting in true negative findings. The future challenge will be understanding if the PET/CT specimen imager might detect micro-metastasis. Furthermore, in patient 1 extra-capsular extension was not suspected in baseline PSMA PET/CT. However, an extra-capsular extension was observed with the specimen imager (and subsequently confirmed by histology), thus hinting at a potential superior sensitivity compared to the whole-body PET images used in clinical practice.

Since surgical margin status is an important prognostic factor in PCa, this is a topic of particular interest. At present, even more deeply studied techniques for surgical margins assessment such as intraoperative frozen section (IFS) for the detection of positive surgical margins in PCa, did not show a significant advantage in terms of oncological outcomes and have still sub-optimal accuracy [13].

In clinical practice, new-generation molecular imaging has already proven its crucial role in the decision-making process of several malignancies, including PCa and NET, and the transition from pre-clinical imaging to clinical practice has been quick and clinically significant. However, new radiopharmaceuticals are emerging beyond PSMA- and SSR-based PET, such as inhibitors of the fibroblast activating protein (FAPi PET) or ligand of the chemokine receptor 4 CXCR4 (Pentixafor PET). All these new radiopharmaceuticals will increase the number of pathways that can be explored with in vivo molecular imaging, and it will increase the understanding of tumor heterogeneity both in primary tumors and in metastatic locations. In this scenario, intra-operative PET/CT specimen imaging offers a link between molecular biology, histopathology assessment, molecular imaging, and radio-guided surgery.

### Limitations and Future Perspectives

This is a pure explorative analysis, as this was the first experience with a PET/CT specimen imager used in prostate cancer and a neuroendocrine tumor in a live-surgery setting. Due to the limited number of patients (n = 3), this analysis only provides only a first indication of the possibilities of intraoperative specimen PET/CT imaging for prostate cancer and neuroendocrine tumors. Extensive future research is needed to further investigate the clinical value of this technique. In this perspective, a deeper understanding of radiotracers’ uptake pattern inside surgical specimens through a PET/CT specimen imager could open newer research pathways that range from imaging (e.g., detection of surgical margins’ involvement) to pathology (e.g., correlation with immunohistochemistry), to clinical oncology (e.g., changes in uptake patterns after treatment). Finally, no comparative analysis with other techniques (e.g., mp-MRI) or an evaluation of the impact on the clinical management of the patients cannot be performed at this stage.

## 5. Conclusions

The use of the PET/CT specimen imager in a live-experience session was feasible and its application has been easily adapted to surgery daily practice. In this pilot analysis, the image acquisition of specimens obtained by patients who received an intra-surgical injection of ^68^Ga-PSMA-11 and ^68^Ga-DOTA-TOC was feasible and reliable, and imaging findings matched perfectly with histopathological analysis. The high sensitivity, together with the evaluation of intra-lesion tumor heterogeneity, was the most relevant result derived from this pilot analysis. Even if far from definitive evidence, this pilot analysis showed promising results that need to be confirmed by further prospective studies in larger cohorts of oncological patients.

## Figures and Tables

**Table 1:** Semi-quantitative data measured both in pre-operative whole-body PET/CT and high-resolution specimen PET/CT images.

	PET/CT SUVmax	Specimen Imager SUVmax
Pt-1 Prostate	26.5	89.2
Pt-1 Left Obturator Nodes	11.4	51.5
Pt-2 Prostate	36.2	57.4
Pt-2 Right Common Iliac Node	11.8	23.2
Pt-2 Left Obturator Nodes	25.9	36.4
Pt-3 Peri-pancreatic Node	52.1	86.6
*SUVmax: Maximum Standardized Uptake Value*		

**Table 2:** Administered activity and acquisition time differences between standard whole-body PET/CT scans and PET/CT specimen imager scans in each patient.

	Administered Activity for Baseline PET	Acquisition Time Baseline PET	Administered Activity for PET/CT Specimen Imager	Acquisition Time PET/CT Specimen Imager
Patient 1	Pt. 1: 160 MBq	60 min	80 MBq	192–273 min
Patient 2	Pt. 2: 180 MBq	60 min	90 MBq	206–275 min
Patient 3	Pt. 3: 175 MBq	60 min	70 MBq	150 min

## Data Availability

Not applicable.
